# UV-visible marker confirms that environmental persistence of *Clostridium difficile *spores in toilets of patients with *C. difficile*-associated diarrhea is associated with lack of compliance with cleaning protocol.e

**DOI:** 10.1186/1471-2334-8-64

**Published:** 2008-05-12

**Authors:** Michelle J Alfa, Christine Dueck, Nancy Olson, Pat DeGagne, Selena Papetti, Alana Wald, Evelyn Lo, Godfrey Harding

**Affiliations:** 1 Department of Medical Microbiology, University of Manitoba, Winnipeg, Manitoba, Canada; 2 Diagnostic Services of Manitoba, Microbiology Discipline, St. Boniface General Hospital site, Winnipeg, Manitoba, Canada; 3 Microbiology laboratory, St. Boniface Research Centre, Winnipeg, Manitoba, Canada

## Abstract

**Background:**

An ultraviolet visible marker (UVM) was used to assess the cleaning compliance of housekeeping staff for toilets in a tertiary healthcare setting.

**Methods:**

The UVM was applied to the toilets of patients who were on isolation precautions due to *Clostridium difficile*-associated diarrhea (CDAD) as well as for patients who were not on isolation precautions. Cleaning was visually scored using a numeric system where 0, 1, 2, and 3 represented; no, light, moderate or heavy residual UVM. Rodac plates containing CDMN selective agar were used to test for the presence of C. difficile on the surfaces of patient's toilets.

**Results:**

Despite twice daily cleaning for the toilets of patients who were on CDAD isolation precautions, the average cleaning score was 1.23 whereas the average cleaning score for toilets of patients not on isolation precautions was 0.9. Even with optimal cleaning (UVM score of 0) *C. difficile *was detected from 33% of the samples taken from toilets of patients with CDAD (4% detection in toilet samples from patients who had diarrhea not due to CDAD).

**Conclusion:**

Our data demonstrated the value of UVM for monitoring the compliance of housekeeping staff with the facility's toilet cleaning protocol. In addition to providing good physical cleaning action, agents with some sporicidal activity against *C. difficile *may be needed to effectively reduce the environmental reservoir.

## Background

Environmental survival of antibiotic resistant organisms (AROs) such as Vancomycin resistant Enterococci (VRE), methicillin resistant *Staphylococcus aureus *(MRSA) and sporulating organisms such as *Clostridium difficile *has been suspected to play a role in nosocomial transmission of these pathogens [1,2]. When patients are diagnosed with infections or as carriers of AROs, they are put on isolation precautions. For some pathogens (e.g. *C. difficile*) the housekeeping cleaning protocols are enhanced in an attempt to reduce the environmental load of these organisms [3]. Reducing the environmental reservoir of these pathogens is thought to reduce the risk of cross-transmission between patients thereby reducing the risk of nosocomial infections caused by these organisms. The current PIDAC guidelines [3] recommend enhanced frequency of cleaning and that if ongoing transmission of *C. difficile *is documented during an outbreak of CDAD in healthcare facilities then 5000 ppm chlorine bleach should be considered for disinfection of the environment (particularly for toilet facilities used by patients with CDAD). However, as outlined by the recent review by Hota [2] it has been difficult to conclusively demonstrate that the presence of this organism in the environment has a causal role in the pathogenesis of nosocomial infections. One of the reasons for this is that the published studies that have evaluated potential interventions aimed at eradicating the ARO from the environmental reservoir were not able to verify compliance of housekeepers with the cleaning protocol. If the housekeeping personnel do not perform the cleaning properly then analysis of the efficacy of environmental interventions cannot be conclusive. A recent study [4] used a UV-visible marker as a means of assessing environmental cleaning. They demonstrated the value of this tool in assessing compliance of housekeeping staff with terminal room cleaning. However, they only assessed whether the marker was removed after 2 to 3 patients had been in the room and it was terminally cleaned when these patients were discharged. There have been no published studies where cleaning was prospectively followed for individual patients.

In North America, nosocomial infections due to *C. difficile *have a higher incidence than all other bacterial gastrointestinal pathogens combined (i.e. *Salmonella species, Shigella species, Campylobacter species, Yersinia, E. coli O157:H7*). Manitoba data for 2002 demonstrated that the combined number of all reported cases of the traditional bacterial gastrointestinal pathogens was 482 cases while there were 936 lab confirmed cases of CDAD for the same time period (data provided by Dr. G. Hammond as part of the "*C. difficile *Surveillance Project Symposium Oct 15, 2003"). In addition there is evidence that the incidence of CDAD in healthcare facilities in many different countries has been increasing over the past 10 years [5-13]. There have been many reports [14,1,2,18] of *C. difficile *spores in the environment of patients who have *C. difficile*-associated disease (CDAD). The spores of this organism are known to survive in the environment for many months [16,1,19,2,20].

The published data suggest that there is a high likelihood that the *C. difficile *spores act as an environmental reservoir that plays a role in nosocomial transmission of this pathogen. The aim of this project was to determine if a UV-visible marker (UVM) could be used to determine the compliance of housekeeping staff with the twice daily cleaning protocols for patients who have been placed on isolation precautions because of CDAD. In addition samples of the toilet were taken to determine if detection of toxigenic *C. difficile *correlated with effectiveness of cleaning the toilets.

## Methods

### Bacterial culture methods

*Clostridium difficile *was grown on Tryptic Soy agar containing 5% sheep blood, vitamin K and hemin (BAK) under anaerobic conditions. All plates were incubated in an anaerobic chamber. To promote sporulation, plates inoculated with *C. difficile *were allowed to have prolonged incubation (usually 7 days) and then the growth was scraped off the agar surface and suspended in sterile reverse osmosis water. The organisms in the suspension were pelleted by centrifugation and washed twice with sterile reverse osmosis water. The suspension was then suspended in 70% alcohol and stored at 4°C until used. Staining by malachite green stain confirmed that the suspension was predominantly spores.

Determination of the concentration of viable spores was performed by serially diluting the spore suspension in sterile phosphate buffered saline and then inoculating 0.1 mLs of each dilution and spreading this over the surface of both BAK agar and CDMN agar (*Clostridium difficile *moxalactam, norfloxacin) agar (Oxoid, Mississauga, ON).

To determine how efficient the Rodac plate method was for sampling spores from surfaces, serial dilutions of *C. difficile *spores were prepared in ATS soil [21]. The spore preparation (0.1 mLs) was inoculated over a toilet seat surface area that was equivalent to the diameter of a Rodac plate and allowed to dry overnight. Rodac plates containing CDMN agar were then pressed onto the surface for approximately 5 seconds. The plates were incubated anaerobically for 48 hours and the colonies counted.

### UV-visible marker (UVM) for cleaning assessment of toilets

**Figure 1:**
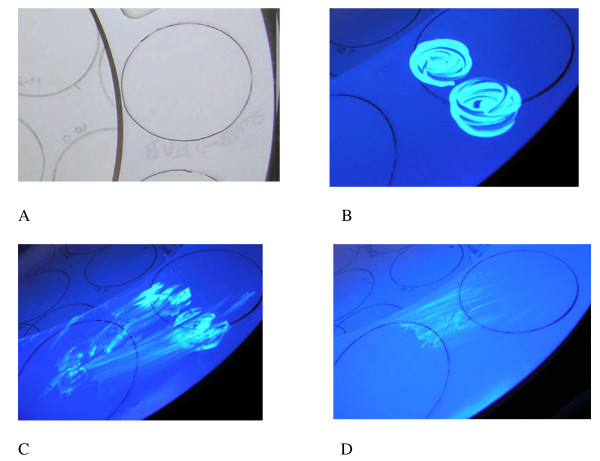
**UV visible marker for verification of toilet cleaning**. Toilet seat lids visualized with regular light (A), and with UV light (B, C, D). The UV marker is scored at 3; shows heavy residual UVM (B), 2; shows moderate residual UVM (C), 1 shows light residual UVM (D), and 0: shows no residual UVM (not shown).

The UVM used for this study was a lotion (Glitterbug^® ^from Brevis Corp., USA). This lotion is non-toxic and water soluble so it is readily removed by cleaning with soap and water solutions. Figure 1 shows how the UVM is not readily visible under regular room lighting but is visible when exposed to short-wave UV light. A hand-held UV light was used for visualization of the marker. The UVM was applied to the underside of the toilet seat or commode and was visually inspected the following day to determine if it had been removed or not. A visual score for residual marker was used; 3 represented heavy fluorescence, 2 represented moderate fluorescence, 1 represented light fluorescence, and 0 represented no fluorescence. Using this numeric scoring system based on visual inspection, an average cleaning score could be determined. Initial testing confirmed that if no cleaning was performed then the UVM showed heavy fluorescence that lasted for at least 7 days after it was inoculated.

### Culture for *C. difficile *from patient-used toilets

Rodac plates containing CDMN agar were used to sample the commodes or toilets in patient rooms. For each toilet (or commode) the agar surface of one plate was sequentially pressed onto the armrest (if present), the underside of the toilet lid (if present), the toilet seat surface, the toilet seat underside, and the inside rim of the upper portion of the toilet bowl. These are all surfaces that should be cleaned as part of regular toilet cleaning. The Rodac plates were then placed into an anaerobic jar and incubated for 48 hours under anaerobic conditions. Suspect colonies were sub cultured to obtain pure cultures and then were confirmed as *C. difficile *based on colony morphology, Gram stain, colony fluorescence under UV light and agglutination using *C. difficile *latex (Microgen Bioproducts, Surrey, UK). The isolates were confirmed as being toxigenic by growing the isolate in Fastidious Anaerobe Broth (FAB) (Lab M Limited, Bury, U.K.) and testing the culture supernatant for Toxin B using the Bartel's CPE assay (Carlsbad, CA). All *C. difficile *isolates were stored as frozen stocks in skim milk at -70°C.

### Housekeeping standard cleaning protocol

Once daily the toilets (and all other high-contact areas in the bathroom) were cleaned and disinfected using PerDiem^® ^(3% Stabilized Hydrogen peroxide from Virox, Mississauga, Canada) at a 1:256 use-dilution (i.e. a final concentration of 0.01% (w/v) stabilized hydrogen peroxide (SHP)). PerDiem^® ^contains 3% (w/v) stabilized hydrogen peroxide as the active agent as well as proprietary "builders and stabilizers." The cleaning protocol consisted of spritzing the SHP solution to wet all of the surfaces of the toilet (or commode) and allowing this to contact the surface while other parts of the bathroom were cleaned. The housekeeping instructions indicate the SHP solution should be allowed to dwell for 10 minutes prior to wiping it off; however, observational assessment of actual practice indicated that the contact time was about three minutes. After about three minutes the toilet was wiped with a cloth rag that had been wet with the same cleaning agent. The cleaning rags were used for one patient toilet only and then were sent for laundering.

### Housekeeping protocol for patients on CDAD Isolation

The cleaning process was the same as indicated above however, each room (including the toilet) was cleaned twice daily (morning and afternoon) and the use-dilution for the PerDiem^® ^was 1:64 (i.e. final concentration of 0.05% SHP).

### Study enrolment

The objective was to compare compliance of housekeepers with the cleaning protocol for toilets in isolation and non-isolation rooms. Ethics and research approval for this study was obtained. Patients were enrolled in one of the following arms of the study:

#### Arm 1

Patients enrolled in Arm 1 of the study had diarrhea, laboratory confirmed CDAD, and were on isolation precautions. The toilets used by these patients were inoculated with UVM each weekday and then visually inspected the next day to determine if the UVM had been removed. Toilets were also sampled each weekday for the presence of detectable *C. difficile *spores using Rodac plates containing CDMN agar. The use-dilution of the 3% SHP cleaning agent was 1:64 (0.05% final SHP concentration) and cleaning was performed twice per day (as per the facility's housekeeping policy). If commodes were used by the patient they were also monitored using UVM and Rodac plates. There may be up to four patients sharing the same toilet facilities.

#### Arm 2

Patients enrolled in Arm 2 of the study had diarrhea, laboratory confirmation that they did not have CDAD and they were not on isolation precautions. The toilets used by these patients were inoculated with UVM each weekday and then visually inspected the next day to determine if the UVM had been removed. Toilets were also sampled each weekday for the presence of detectable *C. difficile *spores using Rodac plates containing CDMN agar. The use-dilution of the 3% SHP cleaning agent was 1:256 (0.01% final SHP concentration), and toilet cleaning was performed once per day (as per the facility's housekeeping policy). If commodes were used by the patient they were also monitored using UVM and Rodac plates. There may be up to four patients sharing the same toilet facilities.

#### Routine Ward cleaning

In addition to the monitoring of toilets as outlined in Arms 1 and 2, routine ward cleaning was assessed by prospectively using UVM to monitor the toilets of all rooms on three separate wards on a daily basis over a 1 week period (Monday to Friday). All toilets were included regardless of whether the patients in the room had diarrhea or not. The toilets should have been cleaned once each day using PerDiem^® ^the SHP cleaning agent at 0.01% final concentration.

As required by the ethics review committee, all housekeeping staff was informed about the study and the use of UVM as a measure of cleaning compliance. However, they did not know which patient rooms would be involved. The results of the marker were not traced back to individual housekeepers and punitive action was not taken even if residual marker was detected.

## Results

### Rodac Plate recovery

The efficiency of *C. difficile *spore recovery by the Rodac plate method was assessed using a spore preparation of known concentration. The Log_10 _average spore inoculum per site was 4.78 (± .51) and the average Log_10 _recovery per site by Rodac plate sampling was 4.94 (± .74).

### UVM Detection

Preliminary testing showed that the 1:256 and 1:64 use-dilutions of the SHP did not interfere with detection of the UVM and only if the marker was physically wiped off was the fluorescence removed (Figure 1). Preliminary testing demonstrated that even the UVM was completely removed with a single wipe using a cloth wet with water (data not shown).

### Prospective monitoring for Arm 1 and Arm 2

There were a total of twenty patients who were followed over a 6 month period between July 2004 and Feb 2005. The toilets and commodes were monitored over the duration of each patient's hospitalization and the results of the UVM and *C. difficile *culture testing are shown in Tables 1 and 2. There were 7 of the 20 patients followed who used commodes at some stage in their hospitalization. The data for the toilet and commode testing (Table 1) demonstrated that there was extremely poor cleaning being performed on the commodes as 72% of the time there was no removal of the UVM.

**Table 1:** Correlation of cleaning efficacy by UVM marker removal and the presence of toxigenic *C. difficile *in patients with CDAD

UVM Cleaning score*	**Number**	Toxigenic *C. difficile *detected** (% of samples with that UVM score)
**Toilets (7 patients; 102 samples)**		

0	52	22 (41.5%)
1	8	2 (8.7%)
2	9	2 (8.8%)
3	33	8 (24.2%)
TOTAL:	102	34 (33.3%)

**Commodes (5 patients; 32 samples)**		

0	4	1 (25%)
1	3	2 (66.7%)
2	2	2 (100%)
3	23	15 (65.2%)
TOTAL:	32	20 (62.5%)

* Cleaning score: Visual inspection was used to assess how much of the UVM remained on the underside of the toilet seat as outlined in the Materials and Methods section.

** Toxigenic *C. difficile *detected: The Rodac culture plate contained at least one colony of *C. difficile *that was confirmed to be toxigenic (i.e. produced Toxin B mediated cytopathic effect using the tissue culture assay).

**Table 2:** Summary of the monitoring for UVM and the presence of toxigenic *C. difficile *in toilets for Arms 1 and 2

Parameter evaluated:	Arm 1 CDAD* (102 samples; 7 patients)	Arm 2 Diarrhea, no CDAD** (99 samples; 13 patients)
Toxigenic *C. difficile *detected at enrolment (%) ***	5/7 (71.4%)	Not applicable
Toxigenic *C. difficile *detected post-enrolment (%)	34/102 (33.3%)	4/99 (4%)
UVM score post-enrolment		
Score 0	51%	58.7%
Score 1	7.8%	6.5%
Score 2	8.8%	20.7%
Score 3	32.4%	14.1%
Average cleaning score post-enrolment	1.2	0.9

*Patients with diarrhea where the diagnostic test for CDAD was positive and the patient was placed on isolation precautions with enhanced housekeeping (twice daily) using 0.05% SHP.

** Patients with diarrhea where the diagnostic test for CDAD was negative and once daily housekeeping was performed using 0.01% SHP.

*** First sample taken before increased frequency of cleaning and use of higher concentration of SHP.

### Prospective monitoring for Routine Ward Cleaning

**Figure 2:**
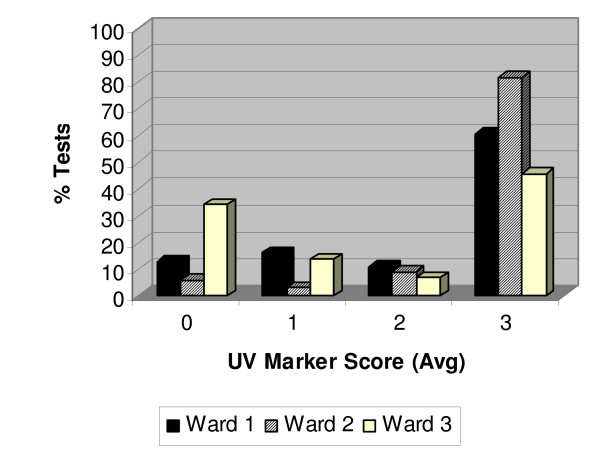
**Routine ward monitoring using the UVM**. Ward 1 (14 rooms) is shown as the solid bar, ward 2 (11 rooms) is shown as the cross-hatched bar, and ward 3 (11 rooms) is shown as the white bar. The toilet in each room was monitored every day prospectively for a week (Monday to Friday). There were 66, 33 and 44 test samples taken from wards 1, 2 and 3, respectively. For ward 2 there was a STAT holiday and samples were not taken on that day so there were 33 samples instead of 44 samples in total.

To further evaluate the UVM as a monitoring tool, the toilets in all patient rooms from three separate wards were monitored. This "routine ward" monitoring provided prospective data on the compliance with routine housekeeping (once per day using 0.01% SHP. The average cleaning score for wards 1, 2 and 3 was 2.1, 2.6, and 1.5, respectively. Stratification of the UVM residuals is shown in Figure 2.

## Discussion

Although the general method was first described by Carling [4], our data is the first to document that by using a UV visible marker it is possible to easily assess the compliance of housekeeping staff with the cleaning protocol for patient toilets. This is a significant finding as it will allow for more accurate analysis of the efficacy of environmental interventions since compliance with cleaning can be verified.

Our data using Rodac plates as a means of monitoring the presence of *C. difficile *on surfaces confirmed that this sample method provides essentially 100% recovery. The count recovered using the Rodac plate was slightly higher than the calculated maximum inoculum but this likely reflects the variability in the method of counting. There have been a number of evaluations of the presence and persistence of *C. difficile *spores in the environment of patients with CDAD. As early as 1981 [14] there was published data demonstrating that the environment of patients with CDAD had a higher likelihood of having *C. difficile *spores compared to those patients who did not have CDAD (9.3% of 910 floor and surface sites for CDAD patients environments compared to 2.6% of 497 similar sites for non-CDAD patients). Environmental contamination with *C. difficile *spores is not surprising as Louie [12] reported that even during and following CDAD treatment patients may shed up to 10^4 ^spores/g feces and Tomiczek [22] commented on the fecal aerosols created when bedpan sprayers are used for cleaning in patient bathrooms. Furthermore, Kim *et al *[14] demonstrated that spores of *C. difficile *could survive for five months on the floor. They also recognized that utilization of contact isolation precautions for CDAD patients was not effective for curbing nosocomial transmission. Our data collected using Rodac plates demonstrates that even after enrolment and implementation of enhanced housekeeping that *C. difficile *can still be detected in the toilets of 33% of CDAD patients.

The value of using the UVM to monitor cleaning compliance of patient toilet facilities was immediately apparent in that it helped identify a major flaw in our housekeeping. The commodes were not being cleaned at all on 72% of the days sampled. This finding was reviewed at a meeting with Infection Control and Housekeeping administration and it was determined that the nursing staff thought the housekeeping staff were responsible for the commodes while the housekeeping staff thought the nurses were responsible for doing it since they helped the patients use the commodes. The responsibility issue was resolved and housekeeping staff were assigned this responsibility.

Carling [4] reported that some degree of UV marker removal was achieved in 80% of toilets that were part of terminal cleaning. However, this is a very crude marker of compliance because it was only done to assess compliance with terminal cleaning (i.e. compliance with daily cleaning was not evaluated). Our prospective daily monitoring indicated that for 32.4% of the days when patients were on CDAD isolation, the toilet cleaning was not done as outlined in the housekeeping policy as the UVM was not removed (UVM score of 3). Patients who were not on isolation precautions appeared to have better toilet cleaning because a score of 3 for the UVM was only present for 14.1% of the samples. The average cleaning score for toilets of patients on isolation precautions was higher (i.e. UV marker not removed as well) than for toilets of patients who were not on isolation precautions. This is surprising as patients on isolation for CDAD were supposed to have had their toilets cleaned twice daily compared to once daily for rooms of patients who were not on isolation precautions. This may be similar to the effect noted by Stelfox *et al *[23] where contact between healthcare staff and patients on isolation precautions is greatly reduced compared to the level of contact for patients who are not on isolation precautions. The lack of cleaning may reflect excess workload for housekeepers or a reluctance to enter rooms of patients on isolation precautions due to inconvenience of following precautions necessary for entering such isolation rooms.

Although the number of patients enrolled in this prospective study was low (7 in Arm 1 and 13 in Arm 2), the number of patient days assessed was 102 in Arm 1 and 99 in Arm 2. Despite the housekeeping policy requiring these rooms to have twice daily cleaning, toilets of CDAD patient rooms had toxigenic *C. difficile *detected on 33% of the days post-implementation of twice-daily cleaning compared to 4% for non-CDAD patient rooms where toilet cleaning was once per day. Our data in Table 1 clearly indicated that the cleaning was suboptimal for the toilets of patients on contact isolation precautions (32.7% with UVM not removed). Even when cleaning was optimal (UVM of 0) there were still high detection rates for toxigenic *C. difficile *(41.5%). Furthermore, our study indicated that *C. difficile *spores were detected on the toilets of CDAD patients over prolonged periods as some toilets still had toxemic *C. difficile *detected on day 28 post-enrolment (data not shown). This suggests that both the physical cleaning action as well as the disinfectant/cleaning agent were ineffective for killing and/or removing *C. difficile *from toilets.

Wilcox *et al *[17] reported that using bleach for environmental disinfection of patient rooms did reduce the incidence of CDAD. As pointed out by Dettenkofer [18], Wilcox's data on the *C. difficile *spores in the environment demonstrated that spores persisted at similar levels regardless of which cleaning/disinfecting agent was used. Although Wilcox [17] documented reduced rates of CDAD this could not be sustained when the wards studied were switched over. These difficulties may well be linked to lack of compliance with the housekeeping protocol. It is impossible to conclusively determine the effect of any housekeeping cleaner/disinfector if the compliance of staff with the physical aspect of cleaning cannot be verified. Although there is some evidence that bleach [16,17] or Accelerated Hydrogen Peroxide [22] can help contain nosocomial spread of CDAD these studies did not attempt to correlate the detection of spores in the environment with the reduction in cases of CDAD. Further studies are needed that use UVM (or some other validated means of assessing cleaning compliance) to correlate the presence of spores in the environment with an intervention using a specific cleaner/disinfector that has activity against *C. difficile *spores.

To determine if the poor compliance with the housekeeping protocol extended to rooms of patients not on isolation precautions regardless of whether they had diarrhea or not, a prospective ward-wide surveillance evaluation was undertaken. The data from this part of the current study (Figure 2) demonstrated that compliance with the routine housekeeping policy was ward dependent. There were dedicated housekeeping staff on each ward therefore; our results likely reflect the compliance of the specific housekeeping staff on each ward. From the initial data it appeared that patients who were on isolation precautions were getting less optimal cleaning compared to the rooms of patients not on isolation. However, the prospective "routine ward" assessment of three other wards for one week indicated that on these wards compliance with cleaning can be even worse than for the isolation rooms. The time of highest risk of transmission of *C. difficile *from one patient to another is likely when the patient is developing diarrhea prior to being diagnosed with CDAD because they have not yet been treated and have not been placed on isolation precautions. As such compliance with routine housekeeping in rooms of patients who are not on isolation precautions is very important because the frequency of physical cleaning is lower and the agent used would have no activity against this organism. Thus use of UVM to monitor compliance to housekeeping protocols would be valuable in all patient rooms – not just those of patients on isolation precautions.

## Conclusion

In summary this study demonstrated the value of using the UVM as a means of monitoring compliance with housekeeping cleaning protocols for all patients regardless of whether they were on isolation precautions or not. Furthermore, UVM monitoring would provide a valuable control for clinical evaluations of intervention agents since the UVM would allow more reliable assessment of housekeeping compliance with the cleaning protocol. We would recommend that UVM monitoring be used on a routine basis as part of the quality assurance program for housekeeping throughout a healthcare facility. Furthermore, our data would support that without an agent with some activity against *C. difficile *spores the physical action of cleaning alone cannot be relied upon to effectively eradicate this organism from the toilets of patients who are shedding this type of spore.

## Competing interests

The authors declare that they have no competing interests.

## Authors' contributions

MJA conceived of the study and participated in its design and coordination and drafted the manuscript. CD, PDeG, EL and GH participated in the design and coordination of the study. NO carried out the data entry and participated in the data analysis. SP, AW and CD performed the toilet testing and participated in the co-ordination of the study. All authors read and approved the final manuscript.

## Pre-publication history

The pre-publication history for this paper can be accessed here:



## References

[B1] Verity P, Wilcox MH, Fawley W, Parnell P (2001). Prospective evaluation of environmental contamination by *Clostridium difficile *in isolation side rooms. J Hosp Infect.

[B2] Hota B (2004). Contamination, Disinfection, and Cross-Colonization: Are Hospital Surfaces Reservoirs for Nosocomial Infection?. CID.

[B3] PIDAC (Provincial Infectious Diseases Advisory Committee) (2006). Best Practices for the Management of Clostridium difficile in all Healthcare Settings.

[B4] Carling PH, Briggs J, Hylander D, Perkins J, Quincy B, Massachusetts S (2006). An evaluation of patient area cleaning in 3 hospitals using a novel targeting methodology. Am J Infect Control.

[B5] Fawley WN, Wilcox MH (2001). Molecular Epidemiology of endemic *Clostridium difficile *infection. Epidemiol Infect.

[B6] Vesta KS, Wells PG, Gentry CA, Stipek WJ (2005). Specific risk factors for *Clostridium difficil*e-associated diarrhea: A prospective, multicenter, case control evaluation. Am J Infect Control.

[B7] Akerlund T, Svenungsson B, Lagergren A, Burman LG (2006). Correlation of Disease Severity with Fecal Toxin Levels in Patients with *Clostridium difficile *– Associated Diarrhea and Distribution of PCR Ribotypes and Toxin Yields In Vitro of Corresponding Isolates. J Clin Microbiol.

[B8] Bartlett JG, Perl TM (2005). The New *Clostridium difficile *– What Does it Mean?. N Engl J Med.

[B9] McDonald LC, Killgore GE, Thompson A, Owens RC, Kazakova SV, Sambol SP, Johnson S, Gerding DN (2006). An Epidemic, Toxin Gene – Variant Strain of *Clostridium difficil*e. N Engl J Med.

[B10] Musher DN, Logan N, Mehendiratta V (2006). Epidemic *Clostridium difficile*. Letter to the Editor. N Engl J Med.

[B11] Wiesen P, Van Gossum A, Preiser JC (2006). Diarrhea in the critically ill. Curr Opin Crit Care.

[B12] Louie TJ (2006). *Clostridium difficile *in clinical practice: Increasing rates, more virulent organisms and new therapies on the horizon. Can J Infect Dis Med Microbiol.

[B13] Hubert B, Loo VG, Bourgault A-M, Poirier L, Dascal A, Fortin E, Dionne M, Lorange M (2007). A portrait of the geographic dissemination of the *Clostridium difficile *North American pulsed-field type 1 strain and the epidemiology of *C. difficile*-associated disease in Quebec. CID.

[B14] Kim KH, Fekety R, Batt DH, Brow D, Cudmore M, Silva J, Waters D (1981). Isolation of *Clostridium difficile *from the environment and contacts of patients with antibiotic-associated colitis. J Infect Dis.

[B15] Griffith CJ, Cooper RA, Gilmore J, Davies C, Lewis M (2000). An evaluation of hospital cleaning regimes and standards. J Hosp Infect.

[B16] Wilcox MH, Fawley WN (2000). Hospital disinfectants and spore formation by *Clostridium difficile*. Lancet.

[B17] Wilcox MH, Fawley WN, Wigglesworth N, Parnell P, Verity P, Freeman J (2003). Comparison of the effect of detergent versus hypochlorite cleaning on environmental contamination and incidence of *Clostridium difficile *infection. J Hosp Infect.

[B18] Dettenkofer M, Hauer T, Daschner FD (2004). Detergent versus hypochlorite cleaning and *Clostridium difficile *infection. Letter to the Editor. J Hosp Infect.

[B19] Block C (2004). The effect of Perasafe^® ^and sodium dichloroisocyanurate (NaDCC) against spores of *Clostridium difficile *and *Bacillus atrophaeus *on stainless steel and polyvinyl chloride surfaces. J Hosp Infect.

[B20] Perez J, Springthorpe S, Sattar S (2005). Activity of selected oxidizing microbicides against the spores of *Clostridium difficile*: Relevance to environmental control. AJIC.

[B21] Alfa MJ, DeGagne P, Olson N (2005). Validation of ATS as an Appropriate Test Soil. Zentr Steril.

[B22] Tomiczek A, Stumpo C, Downey JF (2006). Enhancing patient safety through the management of *Clostridium difficile *at Toronto East General Hospital. Healthcare Quarterly.

[B23] Stelfox HT, Bates DW, Redelmeier DA (2003). Safety of Patients Isolated for Infection Control. JAMA.

